# Constipation prevalence and risk from prescribed medications in people with intellectual disability: Findings from an English mortality programme

**DOI:** 10.1177/17446295241267085

**Published:** 2024-07-19

**Authors:** Christina Roberts, Jonathon Ding, Delia Bishara, Sahar Riaz, Rory Sheehan, Adam White, Andre Strydom, Umesh Chauhan

**Affiliations:** Research Facilitation and Delivery Unit, Applied Health Research hub, 6723 University of Central Lancashire, UK; Institute of Psychiatry, Psychology and Neuroscience, 34426 King’s College London, UK; Institute of Psychiatry, Psychology and Neuroscience, 34426 King’s College London, UK; South London and Maudsley NHS Foundation Trust, UK; 655732 Royal College of Surgeons in Ireland, Ireland; Beaumont Hospital, Dublin; Institute of Psychiatry, Psychology and Neuroscience, 34426 King’s College London, UK; Institute of Psychiatry, Psychology and Neuroscience, 34426 King’s College London, UK; Institute of Psychiatry, Psychology and Neuroscience, 34426 King’s College London, UK; School of Medicine, 6723 University of Central Lancashire, UK

**Keywords:** Intellectual disability, constipation, medication, avoidable mortality

## Abstract

Constipation is common in people with intellectual disability, with case reports of associated deaths. Risk factors include lifestyle factors, health conditions, and certain medications. We aimed to explore constipation in a sample of people with intellectual disability who died in 2021. We described prevalence of constipation, causes of death and the risk of secondary constipation from prescribed medications. Medications were scored based on the risk of constipation indicated in the drug profile. Forty-eight percent of the sample had constipation. Half of the sample were prescribed at least two medications that are commonly associated with side effects of constipation. There were high rates of antipsychotic (30%) and laxative (40%) drug prescription. Five people with a history of constipation died of causes of death associated with constipation. Our findings highlight the risk of secondary constipation due to prescribed medication and the seriousness of the condition in people with intellectual disability.

## Introduction

Constipation is a clinical condition that is defined by ‘infrequent or difficult passage of stool, hardness of stool or incomplete evacuation’ (Bharucha, Pemberton & Locke, 2013). Constipation can be further defined as functional (primary or idiopathic) if the condition is chronic without a known cause, or secondary (organic) if the condition is caused by medication or an underlying medical condition (National Institute for Health and Care Excellence, 2023a).

Constipation is common in people with intellectual disability. Robertson et al. (2017) conducted a systematic review of the prevalence of constipation in people with intellectual disability and found that 14 of 31 studies reported prevalence rates of over 50%. Analysis of the ‘*Learning from lives and deaths – people with a learning disability and autistic people*’ (LeDeR) mortality dataset in England has shown that almost one quarter of people with intellectual disability who died had longstanding constipation (Heslop et al., 2020). A global systematic analysis estimated that the prevalence of constipation in the general population is between 10.1%-15.3% (Barberio, 2021), highlighting the particular risk of constipation amongst people with intellectual disability. The increased risk may be due to numerous factors relating to lifestyle and co-existing health conditions. Risk factors for constipation that are common in those with intellectual disability include obesity (Melville et al., 2007), low levels of exercise (Hawkins & Look, 2006), and higher levels of immobility (Cleaver, Hunter & Oullette-Kuntz, 2009; National Institute for Health and Care Excellence, 2023b).

People with Down syndrome are at particularly high risk of constipation which may be partly due to comorbidity with hypothyroidism (Goday-Arno et al., 2009) and coeliac disease (Du et al., 2018). Increased risk of constipation in people with Down syndrome may also be related to gastrointestinal malformations, hypotonic bowel and increased risk of Hirschprung’s disease (Friedmacher & Puri, 2013; Holmes, 2014).

### Chronic constipation and premature mortality

Constipation may be difficult to detect in people with intellectual disability, which makes it more likely to become a severe or chronic problem. People with intellectual disability may be less likely to recognise the symptoms of constipation and be less able to communicate their symptoms (Coleman & Spurling, 2010). Difficulties describing pain verbally can mean discomfort relating to constipation presents differently; pain can present as behaviours such as food refusal, agitation or self-injury (Doody & Bailey, 2017). Carers may not always be able to recognise constipation in people with intellectual disability because they may not know the signs or may attribute the resulting behaviours to the person’s intellectual disability (Christensen et al, 2009).

Lack of appropriate intervention can lead to chronic constipation and potentially life-threatening complications such as bowel obstruction (Leung, Riutta, Kotecha & Rossa, 2011). There is evidence that constipation is associated with all-cause mortality and cardiac events in the elderly without intellectual disability (Sumida et al., 2019; Judkins et al., 2023; Sundbøll et al., 2020) and exacerbates cognitive decline in people with Parkinson’s disease (Camacho et al., 2021).

The role of constipation in contributing to the premature mortality of people with intellectual disability is not well understood. Constipation has been identified as a major health risk factor for death in people with intellectual disability, particularly aging persons. However, deaths due to constipation are not commonly described in the literature which may be related to the tendency for researchers to use ‘umbrella’ ICD-10 chapters for analysis (‘diseases of the digestive system’), thus obscuring the specific cause of death, or because of issues with the accuracy of cause of death reporting in people with intellectual disability (Maslen et al, 2022). Nevertheless, highly publicised cases of deaths of people with intellectual disability from avoidable complications of constipation have highlighted the urgency of the issue and the importance of obtaining appropriate care (Hill, 2018; Thomas & Cook, 2023).

### Constipation due to side effects of medications

People with intellectual disability may be at risk of secondary constipation due to prescribed medications. Many drugs include constipation as a potential adverse side-effect, such as psychotropics (especially those with anti-cholinergic activity), dietary supplements (e.g. iron and calcium), certain analgesics (e.g. opioids), and anti-seizure medication (Ueki & Nakashima, 2019).

A report by Public Health England investigated the rates and patterns of prescription of psychotropic drugs in people with intellectual disability and autism (Glover et al., 2015). The report found that people with intellectual disability and/or autism have high rates of prescription of all psychotropic drugs and a large proportion of people received longer-term prescriptions for psychotropic medications. Although much of this prescribing may be appropriate, in line with a mental health diagnosis, some may be outside of licensed indication such as in cases of behaviour that challenges (Sheehan et al, 2015). Despite high rates of both constipation and antipsychotic use in those with intellectual disability, the link between constipation and prescribed medications has received little research attention (De Hert et al., 2011; Robertson et al., 2017).

### Treatment of constipation

Best practice guidelines recommend a holistic, individualised approach to bowel management is important for treating constipation (Emly & Rochester, 2006; Emly & Marriott, 2017). This involves clinicians making personalised plans around toileting (for example, advising on posture or adapted seating), exercise and diet (particularly intake of fibre and fluid) and alternative strategies to alleviate symptoms, such as abdominal massage. When these strategies prove ineffective, laxatives are recommended for short-term use and should be regularly reviewed. Despite this, the rate of laxative prescription in people with intellectual disability remains high, with analysis of UK primary care data showing that around 25% of people with intellectual disability are prescribed a laxative at any one point (Carey et al., 2017) and a report showing around 33% of people who died in England with intellectual disability in 2019 were prescribed laxatives (Heslop et al., 2020).

### Summary

In summary, previous research indicates that people with intellectual disability are at increased risk of constipation due to a multitude of factors relating to lifestyle, comorbidities and certain types of prescribed medication. Despite guidance that non-medication treatments are attempted before laxatives are prescribed, laxative prescription remains high in people with intellectual disability. The current work aimed to extend previous literature by estimating constipation-related deaths, use of laxatives and the risk of constipation due to prescribed medication.

## Method

### Data source

This work uses data from the *Learning from lives and deaths – people with a learning disability and autistic people* (LeDeR) programme. LeDeR is a national mortality review programme in England which aims to understand the key issues facing the health of people with intellectual disability by identifying a range of topics for further review. LeDeR has received international recognition for collecting rich data on the lives and deaths of people with intellectual disability.

Deaths of people with intellectual disability are reported to the programme via the LeDeR website. If a notification of a death is found to be in the scope of the programme (the person has a confirmed intellectual disability and died whilst being a registered health service user in England), the death will be reviewed by a trained reviewer.

The programme conducts two types of reviews of deaths, initial and focused reviews. The information in initial reviews is limited to demographic information and information about the death. Focused reviews are conducted when reviewers consider significant learning could be gained from looking in further detail at a death or a death is within scope of local health service priorities, for example, some NHS areas look at all deaths of people who died from pneumonia. Deaths of people from ethnic minority backgrounds are automatically forwarded for a focused review. Reviewers collect more extensive information, gathered from the medical record and through interviews with clinicians and caregivers. This report included information from focused reviews.

Information available included age at death, sex, ethnicity, level of intellectual disability (mild, moderate, severe and profound/multiple), Down syndrome impaired mobility (defined as any mobility impairment, from relying on a walking aid to being bedbound) and living situation (residential or nursing home, supported living, family home, rented, other). In England, supported living for vulnerable adults involve placements in rented accommodation with support from social and/or health care staff to enable independence for those who have care needs (National Health Service, 2023). ‘Other’ living situation includes circumstances which do not fit into the categories, for example, if someone is transitioning between two different living situations.

Long-term conditions are defined as “acquired conditions that cannot be cured but can be controlled with ongoing management using medication and/or other therapies over a period of years” (White et al., 2022). People were recorded as having constipation or a long-term condition (including physical health conditions: dysphagia, sensory impairment, respiratory problems, epilepsy, cardiovascular conditions, diabetes, musculoskeletal problems, cancer, dementia, kidney problems, hypertension, and obesity; and metal health conditions: depression, anxiety, other mental health need (e.g. obsessive compulsive disorder), psychosis, bipolar affective disorder) if they had a clinical diagnosis in their medical record.

### Sample

Of the total 139 adults who had died and had a focused review completed in 2021, we selected only those who had medication information available. A total of 96 people who died in 2021 and had a focused review that was completed in the same year were therefore included in this analysis.

People with constipation were defined as those with a diagnosis of constipation (including acute and chronic constipation) at their time of death in their medical record.

### Cause of death

To investigate causes of death, we used information from the death certificate completed by a registered medical practitioner. The death certificate (known as the Medical Certificate of Cause of Death in England) lists the sequence of events leading to the death, including co-occurring conditions. To report the cause of death in this report, we used the underlying cause of death which is defined as the disease or condition that was responsible for the death.

In order to explore whether an underlying recorded cause of death was likely to have been associated with (the condition could have caused constipation) or contributed to (constipation could have contributed to the condition) by constipation one of the authors, who is a medical doctor, reviewed all the underlying causes of death relating to diseases of the digestive system.

### Laxative use

To assess laxative use, each medication a person was prescribed at their time of death were coded as 0 (non-laxative medication) or 1 (laxative medication), according to a list of laxative medications (see Table 1) compiled by the authors using guidance from the British National Formulary (National Institute for Health and Care Excellence, n.d.a). The total score for each person was calculated and reported as a ‘laxative score’, equating to the number of different laxative medications a person was prescribed at the time of their death. Laxatives that were only prescribed in the week before the death were not counted as these were not considered to be part of someone’s routine medication. We were only able to account for prescribed laxative medications and over-the-counter laxatives were not included.

**Table 1.** Types of laxatives.

Medication name examples	Type
Senna	Stimulant laxatives
Macrogol (Movicol, Laxido)	Osmotic laxatives
Lactulose	Osmotic laxatives
Docusate	Stool softener laxatives
Glycerol	Suppository/enema
Sodium acid phosphate with sodium phosphate	Enema
Prucalopride	Prokinetic laxatives
Ipsaghula husk	Bulk-forming laxatives

### Antipsychotic use

To estimate the number of people in the sample who were prescribed anti-psychotics, the authors reviewed each medication a person was prescribed at their time of death. If the person was prescribed any antipsychotic drug listed on the British National Formulary catalogue of drugs (National Institute for Health and Care Excellence, n.d.b) they were on antipsychotics.

### Risk stratification of other prescribed medication

To assess the risk of constipation arising from other prescribed medications, the medication record for each review was compared to a list of medications with constipation as a side-effect compiled by a pharmacist and a medical doctor (Bishara et al., 2023). The list was developed by collating medications in the British National Formulary (BNF) that list constipation in the drug side-effect profile. It included 535 medications, which were grouped based on the summary of product characteristics for each drug (which is based on drug trials and indicate the likelihood of people taking the medication to experience constipation) and given a score. The groupings were as follows:

- A common or very common side-effect: An estimated prevalence between 1 in 10 and 1 in 100 people taking the medication. Constipation risk score = 4.
- An uncommon side-effect: An estimated prevalence between 1 in 100 and 1 in 1,000 people taking the medication. Constipation risk score = 3.
- A rare or very rare side-effect: An estimated prevalence between 1 in 1,000 and 1 in 10,000 people taking the medication. Constipation risk score = 2.
- Medications associated with constipation in certain circumstances or when administered with other drugs were scored 1.

Medications which were not listed were scored as 0 (not known) as they are not associated with constipation. The medications recorded in the 96 people in the sample were reviewed and scored according to the above scoring system. To ensure accuracy, two researchers checked each other’s scoring. Constipation risk was calculated by adding together the risk scores of every medication a person was prescribed at the time of their death – we termed this the prescribed medication constipation risk score.

### Statistical analysis

Multiple logistic regression was used to determine the factors that predict constipation. The independent variables were age, sex, ethnicity, level of intellectual disability, impaired mobility, Down syndrome, living situation and number of long-term conditions.

Multiple linear regression was used to assess the factors that predict risk that prescribed medications were associated with constipation (see risk stratification), which was the dependent variable. The independent variables were age, sex, ethnicity, level of intellectual disability, impaired mobility, Down syndrome, living situation and number of long-term conditions. These were the same variables as used in the multiple logistic regression.

The independent variables for regression analyses were tested for multicollinearity and the Variance Inflation Factors (VIF) were all below 2.62. Linearity between the independent and dependent variables were plotted and no evidence of nonlinear relationships was visualized. Sampling distributions were produced using 2,000 bootstraps with replacement.

All statistical analyses were conducted using Stata 18 and reported based upon a significance level of 5% and 95% confidence intervals.

## Results

### Demographics

Of the 96 people in the overall sample, 59.4% (n = 57) were male. The median age at death of the overall sample was 56.5 years. The overall sample was mostly white (72.9%, n = 70). Around a third had a moderate intellectual disability (33.3%, n = 32) or severe intellectual disability (33.3%, n = 32), 16.7% (n = 16) had mild and 8.3% (n = 8) had a profound/multiple intellectual disability. Around nineteen percent of the overall sample had Down syndrome. The majority (68.8%, n = 66) of the sample had impaired mobility. The most common place of residence was residential/nursing home (41.7%, n = 40).

Forty-six (47.9%) people in the sample had a clinical diagnosis of constipation. The median age at death of the subsample with constipation was 57 years. The demographics of the subsample of people with constipation alongside the overall sample is shown in Table 2.

**Table 2.** Demographics of the overall sample and subset of the sample with constipation.

Variable	Frequency (%) of the overall sample (N = 96)	Frequency (%) of the sample with constipation (N = 46)
Age:		
18-19	5 (5.2%)	2 (4.4%)
20-29	5 (5.2%)	1 (2.2%)
30-39	11 (11.5%)	3 (6.5%)
40-49	10 (10.4%)	5 (10.9%)
50-59	23 (24.0%)	9 (19.6%)
60-69	22 (22.9%)	15 (32.6%)
70-79	16 (16.7%)	9 (19.6%)
80+	4 (4.2%)	2 (4.4%)
Sex:		
Male	57 (59.4%)	27 (58.7%)
Female	39 (40.6%)	19 (41.3%)
Ethnicity:		
White	70 (72.9%)	31 (67.4%)
Black, African, Caribbean or Black British	9 (9.4%)	5 (10.9%)
Asian or Asian British	8 (8.3%)	3 (6.5%)
Mixed	5 (5.2%)	1 (2.2%)
Other	2 (2.1%)	4 (8.7%)
Level of intellectual disability:		
Mild	16 (16.7%)	3 (6.5%)
Moderate	32 (33.3%)	16 (34.8%)
Severe	32 (33.3%)	17 (37.0%)
Profound-multiple	8 (8.3%)	6 (13.0%)
Not known	8 (8.3%)	4 (8.7%)
Down syndrome	18 (18.8%)	5 (10.9%)
Impaired mobility	66 (68.8%)	35 (76.1%)
Living situation:		
Residential or nursing home	40 (41.7%)	20 (43.5%)
Supported living	22 (22.9%)	14 (30.4%)
Family home	21 (21.9%)	8 (17.4%)
Rented	5 (5.2%)	2 (4.3%)
Other	8 (8.3%)	2 (4.3%)

### Long-term conditions

The median number of co-morbid long-term conditions for each person in the overall sample was 2 (IQR = 2). The five most common comorbidities were: sensory impairments (50.0%, n = 48), dysphagia (42.7%. n = 41), respiratory conditions (38.5%, n = 37), epilepsy (38.5%, n = 37) and cardiovascular conditions (32.3%, n = 31). The proportion of the sample with at least one mental health condition was 41.7% (n = 41). Fifteen percent (n = 14) were obese (BMI >30kg/m^2^).

The median number of co-morbid long-term conditions each person with constipation (n = 46) had in addition to intellectual disability was 2 (IQR = 2). The long-term conditions of the overall sample and the subset of the sample with constipation are in Table 3.

**Table 3.** Long-term conditions of the overall sample and the subset of the sample with constipation.

Long-term condition	Frequency (%) of the overall sample (N = 96)	Frequency (%) of the sample with constipation (N = 46)
Physical health:		
Dysphagia	41 (42.7%)	26 (56.5%)
Sensory impairment	48 (50.0%)	24 (52.2%)
Respiratory problems	37 (38.5%	18 (39.1%)
Epilepsy	37 (38.5%)	20 (43.5%)
Cardiovascular (excl. hypertension)	31 (32.3%)	12 (26.1%)
Diabetes	25 (26.0%)	8 (17.4%)
Musculoskeletal problems	23 (24.0%)	17 (37.0%)
Cancer	16 (16.7%)	10 (21.7%)
Dementia	13 (13.5%)	7 (15.2%)
Kidney problems	12 (12.5%)	7 (15.2%)
Hypertension	11 (11.5%)	1 (2.2%)
Obesity (BMI >30kg/m2)	14 (14.6%)	3 (6.5%)
Mental health:		
Depression	25 (26.0%)	14 (30.4%)
Anxiety	14 (14.6%)	11 (23.9%)
Other mental health need	10 (10.4%)	3 (6.5%)
Psychosis	9 (9.4%)	7 (15.2%)
Bipolar affective disorder	2 (2.1%)	0
Total number of people with any mental health condition recorded	40 (41.7%)	22 (47.8%)
Summary statistics		
number of long-term conditions (IQR)	2 (2)	2 (2)

### Causes of death in people with constipation

Of the 46 people in the subsample who were recorded as having constipation, none had constipation recorded on any part of their cause of death record. The most common disease or condition listed as the primary cause of death was pneumonia (encompassing a range of types of pneumonia such as aspiration pneumonia and including COVID-19 pneumonitis), representing 39.1% (n = 18) of people who died.

Nine (20%) people in the subsample of people with constipation had conditions that are associated with constipation on their death certificate (e.g. bowel cancer, gastroparesis and ulcerative colitis).

Three had conditions that could be contributed to by constipation (spontaneous intestinal obstruction, sigmoid volvulus and sigmoid perforation) as the most immediate cause of death. Two people had conditions that could be caused by or contributed to by constipation listed in the sequence of conditions which lead directly to death (perforation of sigmoid volvulus, bowel obstruction). None of the 50 people in the overall sample without constipation had causes of death associated with constipation recorded in any part of their death certificates.

### Laxatives

Of the 96 people in the overall sample, 37.5% (n = 36) were prescribed laxatives at the time of their death. Of the subsample with constipation (n = 46), 63.0% (n = 29) were prescribed laxatives and 37.0% (n = 17) were not prescribed laxatives.

Of the people who were prescribed laxatives (n = 36), most were prescribed one laxative medication (51.3%, n = 18); over a third (35.9%, n = 13) were prescribed two laxative medications, and 12.8% (n = 5) were prescribed 3 different laxative medications.

### Antipsychotic medication

Of the 96 people in the overall sample, 29 people (30.2%) were prescribed antipsychotics at the time of death. Sixty-seven people (69.8%) were not prescribed antipsychotics.

Of the 46 people with constipation, 15 people (32.6%) were prescribed antipsychotics at the time of death. Thirty-one people (67.4%) were not prescribed antipsychotics.

### Risk of constipation from medication

The median prescribed medication constipation risk score was 8 (IQR = 8). This means that 50% of the overall sample obtained a constipation risk score that was equivalent to being prescribed two or more medications that are commonly or very commonly associated with constipation. A constipation risk score that is equivalent to being prescribed three or more medications that are commonly or very commonly associated with constipation was obtained by 20% of the overall sample.

### Correlation between number of prescribed laxatives and constipation risk score

There was a small positive correlation (r = 0.38) between the constipation risk score and the number of laxatives someone was prescribed.

### Regression analyses

#### Factors that predict constipation

Logistic regression was used to determine factors associated with constipation. The independent variables were age, sex, ethnicity, level of intellectual disability, impaired mobility, Down syndrome, living situation and number of long-term conditions.

Several of the variables were associated with higher odds ratios (OR) for constipation in the adjusted analysis (see Table 4). For example, increasing age (in years) was associated with an odds ratio for constipation of 1.06 (95% CI 1.02, 1.10). A person’s level of intellectual disability was a predictor of constipation after taking account of the other variables in the adjusted analysis. Having profound/multiple disability was associated with an odds ratio for constipation (OR 6.99, 95% CI 1.30, 37.71). Down syndrome was the only variable in the adjusted analysis that was associated with a lower odds ratio for constipation (OR 0.17, 95% CI 0.04, 0.72).

**Table 4.** Results of a logistic regression exploring factors predicting constipation.

Variable	With constipation (N, % of total)	Without constipation (N, % of total)	Overall total (N, %)	Adjusted Odds Ratio	Confidence interval
Age (in years)	46 (100%)	50 (100%)	96 (100%)	1.06	1.02,1.10
Sex:					
Female	19 (41.3%)	20 (40.0%)	39 (40.6%)	**-**	**-**
Male	27 (58.7%)	30 (60.0%)	57 (59.4%)	0.96	0.35,2.64
Ethnicity:					
White	31 (67.4%)	39 (78.0%)	70 (72.9%)	**-**	**-**
Minority Ethnic background	15 (32.6%)	11 (22.0%)	26 (27.0%)	2.63	0.82,8.43
Level of intellectual disability					
Mild	3 (6.5%)	13 (26.0%)	16 (16.7%)	**-**	**-**
Moderate	16 (34.78%)	16 (32.0%)	32 (33.3%)	3.43	0.69,17.18
**Severe/Profound/multiple**	**23 (50.0%)**	**17 (34.0%)**	**32 (33.3%)**	**6.99**	**1.30,37.71**
Not known	4 (8.7%)	4 (8.7%)	8 (8.33%)	4.86	0.51,46.29
Impaired mobility:					
Unimpaired mobility	11 (23.9%)	19 (38.0%)	40 (41.7%)	**-**	**-**
Impaired mobility	35 (76.1%)	31 (62.0%)	66 (68.8%)	2.33	0.74,7.39
Down syndrome:					
No down syndrome	41 (89.1%)	37 (74.0%)	78 (81.3%)	**-**	**-**
**Down syndrome**	**5 (10.9%)**	**13 (26%)**	**18 (18.8%)**	**0.17**	**0.04,0.72**
Living situation:					
Residential or nursing home	20 (43.5%)	20 (40.0%)	41.7%	**-**	**-**
Supported living	14 (30.4%)	8 (16.0)	22 (22.9%)	1.30	0.36,4.71
Family home	8 (17.4%)	13 (26.0%)	21 (21.9%)	1.31	0.34,5.06
Other	4 (8.7%)	9 (18.0%)	13 (13.5%)	0.23	0.03,1.62
Number of long-term conditions	46 (100%)	50 (100%)	96 (100%)	1.04	0.70,1.55

Sex, ethnicity, impaired mobility, living situation and the number of long-term conditions were not associated with having constipation. Level of learning disability and living situation did not have significant contributions to the model as indicated by insignificant Wald test statistics (Level of learning disability: Wald = 1.06, p = 0.37; living situation: Wald = 0.65, p = 0.69).

#### Factors that predict risk that prescribed medications were associated with constipation

A bootstrapped multiple linear regression was used to assess the factors that predict risk that prescribed medications were associated with constipation (see risk stratification), which was the dependent variable. The independent variables were age, sex, ethnicity, level of intellectual disability, impaired mobility, Down syndrome, living situation and number of long-term conditions.

The overall model explained 22% of the variance in constipation risk score. (see Table 5). There were two independent variables that demonstrated a significant association with prescribed medication constipation risk score: number of long-term conditions and down syndrome (see Table 6). The observed coefficient for the number of long term conditions was 2.01 (SE = 0.54; p < 0005) which indicates that on average someone’s constipation risk score increases by approximately 2.01 points for each long-term condition they have due to medication they have been prescribed. The observed coefficient for down syndrome was -3.37 (SE = 1.64; p = 0.04).

**Table 5.** Overall regression output for the model predicting risk that prescribed medications were associated with constipation.

Linear regression	
Number of observations	96.00
Replications	2,000
Wald (chi^2^)	33.89
Prob > chi^2^	0.0007
R-squared	0.22
Adjusted R^2^	0.11
Root MSE	7.71

**Table 6.** Factors predicting risk that prescribed medications were associated with constipation.

Variable	Observed coefficient	Standard error	t value	P value	Confidence interval
Age (years)	0.06	0.05	1.12	0.27	-0.04, 0.16
Male (reference = female)	2.33	1.62	1.44	0.15	-0.85, 5.52
British minority ethnic background (reference = white)	-0.18	1.93	-0.09	0.93	-3.95, 3.60
Level of intellectual disability (reference = mild)					
Moderate	0.32	2.47	0.13	0.90	-4.51, 5.15
Severe/Profound/Multiple	0.48	2.43	0.20	0.84	-4.29, 5.25
Not known	-0.41	3.36	-0.12	0.90	-7.00, 6.18
Impaired mobility (reference = unimpaired mobility)	1.94	1.99	0.97	0.33	-1.96, 5.85
**Down syndrome**	**-3.37**	**1.64**	**-2.05**	**0.04**	**-6.58, -0.15**
Living situation					
Supported living	4.41	2.40	1.84	0.07	-0.29, 9.10
Family home	1.56	2.31	0.68	0.50	-2.96, 6.09
Other	-0.33	2.32	-0.14	0.89	-4.88, 4.21
**Number of long-term conditions**	**2.01**	**0.54**	**3.73**	**<0.005**	**0.95, 3.07**
constant	-1.86	3.82	-0.49	0.63	-9.35, 5.64

### Discussion

The aim of this report was to provide an in-depth investigation of constipation in people with intellectual disability who died in 2021. There are three main findings. The first is that a large portion of the sample were at risk of developing secondary constipation as a result of their prescribed medications. The second is that laxative prescription was very common, with 40% of the people with intellectual disability included in this sample being prescribed at least one laxative. The third main finding is that constipation did not appear on any of the death certificates for the people included in this sample either as a contributory factor or more proximal cause of death, however, of those recorded as having constipation, six (13.0%) died of bowel obstruction or perforation. In comparison, none of the people without constipation died of bowel perforation or obstruction, suggesting that constipation may be related to deaths despite not being formally recorded.

To our knowledge, this is the first time that the prescribed medications of people with intellectual disability have been reviewed collectively to determine the composite risk of developing constipation as a side-effect. We found that half of the sample had a score that equated with taking two or more medications commonly or very commonly associated with constipation. One-fifth obtained a score that was equivalent to taking four or more medications commonly or very commonly associated with constipation. Prescriptions of anti-epileptic and antipsychotic medications, which are both frequently associated with constipation, may have contributed to the elevated constipation risk scores observed in this sample. The overall sample contained a high proportion (43.5%) of people with epilepsy, which is greater than the estimated prevalence of 22% in people with intellectual disability (Robertson et al., 2015). Additionally, over 30% of the sample were prescribed antipsychotics at their time of death, in keeping with existing data that has shown high rates of antipsychotic medication use in people with intellectual disability (Glover et al., 2015). For comparison, it is estimated that less than 1% of the general population have epilepsy (Bell, Neligan & Sander, 2014) and around 1% are prescribed antipsychotics (Shoham et al., 2021).

Logistic regression was used to explore the factors predicting constipation. There were three variables associated with higher odds ratios for constipation: increasing age and having a severe or profound level of intellectual disability. Having Down syndrome was the only variable in the adjusted analysis that was associated with a lower odds ratio for constipation, although there were only a small number of people in the sample who had Down syndrome and this finding needs to be interpreted with caution. Some of these results support findings from a systematic review which demonstrated that increasing age and profound intellectual disability are associated with constipation (Robertson et al., 2017). However, the finding that Down syndrome was associated with a lower odds ratio for constipation contrasts with results which identified Down syndrome as a risk factor for constipation (Robertson et al., 2017).

Immobility has been identified as a risk factor for constipation. However, immobility was not associated with a higher or lower risk of constipation in this sample, perhaps because it only included people who have died and thus frailer than the community-dwelling population of people with intellectual disability. Levels of mobility could have been affected by the COVID-19 pandemic, which saw many people with a learning disability still shielding and confined to their homes as a highly vulnerable group to COVID-19 infection (Courtenay and Cooper, 2021).

Multiple regression analyses were used to explore the factors associated with the likelihood of being prescribed medications that are more likely to cause constipation. Only number of long-term health conditions was associated with likelihood of being prescribed medications that have constipation as a side-effect. This finding is unsurprising as people who have several health conditions are more likely to be prescribed a greater number of medications to treat these conditions, some of which may be associated with constipation. Several variables including age, sex, ethnicity, level of intellectual disability, impaired mobility, Down syndrome and living situation were not significantly associated with the risk that prescribed medications were associated with constipation. Although our findings suggest that age, sex and reduced mobility are not associated with the risk of being prescribed medications, this is likely because they are not, of themselves, health conditions requiring medication. Instead, health conditions related to these factors (for example, frailty in old age) may be associated with increased risk that prescribed medications were associated with constipation. A high proportion (40%) of the sample were prescribed laxatives. The high rates of laxative prescriptions found in this sample have been shown previously using data from a mortality review, which found 33% of people with intellectual disability were prescribed laxatives (Heslop et al., 2020). Laxative prescription only had a weak correlation with constipation risk score. In addition, 37% of people who had constipation were not prescribed laxatives. However, as our analysis does not account for over-the-counter laxative use, it could be that the actual rate of laxative use is higher than reported.

A high proportion of the sample resided in supported living or family homes with less intensive nursing input, so may have been more likely to use over-the-counter options for convenience rather than obtaining a prescription. It could also be the case that people with constipation were following plans of non-medication treatment for constipation, however we did not have the information to confirm this. The high rate of laxative prescription in this sample may be due to the significant number of people with impaired mobility. People with a learning disability with impaired mobility may have been less likely to have been prescribed non-pharmacological treatments for constipation as many non-medication treatments rely on the ability to mobilise.

Five deaths had constipation-related conditions on their death certificate, such as sigmoid volvulus and bowel obstruction. These findings add to existing literature suggesting that chronic constipation could contribute to premature mortality in people with intellectual disability (Cooper et al., 2020).

### Strengths and limitations

To our knowledge, this report is the first to quantify the risk of secondary constipation in a sample of people with intellectual disability based on their prescription medication. The use of a validated list developed to assess constipation risk score ensured the risk stratification process was systematic and based on available evidence from the side effects listed in drug profiles. By using data from a mortality review programme, we were able to add to the available evidence about constipation-related deaths in people with intellectual disability.

A limitation of this report is the sample of people who died that was used in this report is a subset (n= 96) group of people died in 2021 and were reported to a national mortality programme in England. These reviews are only conducted in certain circumstances (e.g. where there are concerns about a death) and the sample may therefore not be representative of the wider group of people with intellectual disability.

The overall number of people in this report was relatively small which means that it may be underpowered. We did not have information about length of prescription for any medications or details of medication reviews to assess the extent to which prescription of laxatives and psychotropics adhere to best practice clinical guidelines.

### Implications

People with intellectual disability should be provided with accessible advice and education about diet and lifestyle measures that can prevent constipation. Education about normal bowel function should be offered to people with intellectual disability and their caregivers, particularly those who are older and have severe-profound intellectual disability who are more at risk of constipation and those who are prescribed medication that could affect bowel function, or with known bowel conditions (Public Health England, 2016).

To mitigate the constipating effects of medications, regular medication reviews should be undertaken, particularly for individuals with multimorbidity and polypharmacy. These reviews should be holistic, accounting for the multitude of risk factors those with intellectual disability are already predisposed to for developing constipation in prescribing decisions, and reviews should be conducted by a clinician experienced in intellectual disability. To assist with this, the Maudsley BRC has developed an app which can be used to identify medications with high risk of causing constipation (Bishara et al., 2023). The app could be used routinely at medication review meetings and consulted before adding new medications to an individuals’ regimen.

In those at high risk for developing constipation, bowel monitoring is essential to ensure constipation is identified and treated early before laxatives become necessary. Further work is needed to develop resources to support individuals involved in the care of people with intellectual disability to recognise, escalate and manage constipation.

Future research should validate the prescribed medication and constipation risk stratification procedure in community samples of people with intellectual disability to assess the risk of constipation more comprehensively from medications. Using statistical modelling to account for other potential risk factors of constipation would allow more reliable estimates of risk of constipation based on medication in this population. Other data sources could be accessed to better understand the age at which people with intellectual disability develop constipation and how non-medication treatments are implemented. Research investigating prescribing practices for laxatives and psychotropics would be useful in establishing how well current practice adhere to guidelines.

### Conclusion

The findings demonstrate that constipation is a significant issue for people with intellectual disability. The prevalence rate for constipation of 48% is consistent with previous research which has estimated the rate of constipation to be between 25% and 50% in this population (Robertson et al., 2017). By quantifying the risk of constipation from prescribed medications, our findings suggest people with intellectual disability may be at risk of secondary constipation and highlight the importance of considering this when making prescribing decisions.
